# Neurologically Intact Recovery After Extracorporeal Cardiopulmonary Resuscitation for Cardiac Arrest Due to Blow-Out-Type Left Ventricular Free Wall Rupture: A Case Report

**DOI:** 10.7759/cureus.117801

**Published:** 2026-10-12

**Authors:** Kazuki Morooka, Yuka Higuchi, Shunya Ono, Kosaku Nishigawa, Takeyuki Kanemura

**Affiliations:** 1 Cardiovascular Surgery, IMS Katsushika Heart Center, Tokyo, JPN

**Keywords:** acute myocardial infarction, blow-out-type rupture, cardiac arrest, extracorporeal cardiopulmonary resuscitation, left ventricular free wall rupture, mechanical circulatory support, neurologically intact survival, venoarterial extracorporeal membrane oxygenation

## Abstract

Blow-out-type left ventricular free wall rupture after acute myocardial infarction is a catastrophic complication that often causes cardiac arrest. Neurologically intact survival after extracorporeal cardiopulmonary resuscitation is rare, particularly when extracorporeal support must be established outside a catheterization laboratory. We report a patient who survived neurologically intact despite the initiation of venoarterial extracorporeal membrane oxygenation without fluoroscopic guidance and with markedly limited blood flow.

A 74-year-old woman with acute myocardial infarction developed cardiac arrest due to blow-out-type left ventricular free wall rupture while awaiting emergency coronary angiography. Cardiopulmonary resuscitation was initiated immediately, and venoarterial extracorporeal membrane oxygenation was established by femoral cannulation without fluoroscopic guidance 16 min after cardiac arrest. Cardiac tamponade limited extracorporeal blood flow to approximately 1.5 L/min. She was transferred directly to the operating room, where blow-out rupture of the left ventricular apex was confirmed. After conversion to cardiopulmonary bypass, the rupture was repaired with felt strips, and coronary artery bypass grafting to the left anterior descending artery was performed using a saphenous vein graft. She regained consciousness without apparent neurological deficits, was extubated on postoperative day three, and was transferred to a rehabilitation hospital on postoperative day 49. At 12 months, she remained clinically well. Rapid diagnosis, extracorporeal cardiopulmonary resuscitation, and immediate surgical repair may enable neurologically intact survival after cardiac arrest caused by blow-out-type left ventricular free wall rupture.

## Introduction

Blow-out-type left ventricular free wall rupture (LVFWR) is a catastrophic mechanical complication of acute myocardial infarction that can cause abrupt hemopericardium, cardiac tamponade, profound circulatory collapse, and sudden cardiac arrest [1]. Unlike more contained forms of LVFWR, blow-out rupture is characterized by active communication between the left ventricular cavity and the pericardial space, resulting in rapid blood loss and hemodynamic deterioration that generally requires immediate surgical repair.

Although extracorporeal cardiopulmonary resuscitation (ECPR) may provide temporary circulatory support in patients with refractory cardiac arrest, its effectiveness can be markedly limited in the presence of cardiac tamponade because impaired venous return may result in insufficient drainage and inadequate extracorporeal blood flow. This creates a particularly challenging situation in which extracorporeal support may be necessary to maintain circulation, while definitive survival still depends on prompt surgical decompression and repair of the rupture.

Survival with a favorable neurological outcome after cardiac arrest caused by blow-out-type LVFWR has rarely been reported. In several previously reported survivors, extracorporeal support was established in a catheterization laboratory, or the patient was rapidly transferred there during resuscitation, allowing immediate access to fluoroscopy, mechanical circulatory support, coronary angiography, and subsequent surgical treatment [2-4].

We report a case of blow-out-type LVFWR following acute myocardial infarction that caused cardiac arrest in the emergency department. Although venoarterial extracorporeal membrane oxygenation (VA-ECMO) was initiated without fluoroscopic guidance and extracorporeal blood flow remained severely limited because of cardiac tamponade, the patient underwent immediate surgical repair and survived without neurological impairment.

## Case presentation

The patient was a 74-year-old woman with a medical history of hypertension and rheumatoid arthritis. She also had untreated hypercholesterolemia and chronic kidney disease. She had no history of diabetes mellitus or smoking. She had experienced intermittent chest discomfort for approximately one month but had not sought medical attention because the symptoms were mild and self-limiting. On the day of admission, she developed acute-onset chest pain in the early morning and presented to a nearby clinic. Electrocardiography (ECG) revealed ST-segment elevation, and she was transferred to our institution with a diagnosis of acute myocardial infarction.

Upon arrival, she was alert and hemodynamically stable. Her blood pressure was 150/80 mmHg, heart rate was 90 beats/min with sinus rhythm, and oxygen saturation was 99% while breathing ambient air. Continuous monitoring was initiated in the emergency department while preparations for emergent coronary angiography were underway. Blood tests, ECG, and transthoracic echocardiography were performed concurrently.

ECG showed QS complexes in leads V1 and V2 and ST-segment elevation in leads V1-V3, consistent with an extensive myocardial infarction in the left anterior descending (LAD) artery territory (Figure 1). Transthoracic echocardiography similarly demonstrated akinesis of the anteroseptal wall extending from the papillary muscle level to the apex, with thinning and aneurysmal dilatation of the apical myocardium. These findings suggested a large anterior myocardial infarction as the underlying cause of the subsequent ventricular rupture. No pericardial effusion was observed at that time. Laboratory testing showed a creatine kinase-MB level of 257 U/L, a cardiac troponin I level of 65.34 ng/mL, a serum creatinine level of 1.27 mg/dL, and an estimated glomerular filtration rate of 32.1 mL/min/1.73 m^2^.

**Figure 1:**
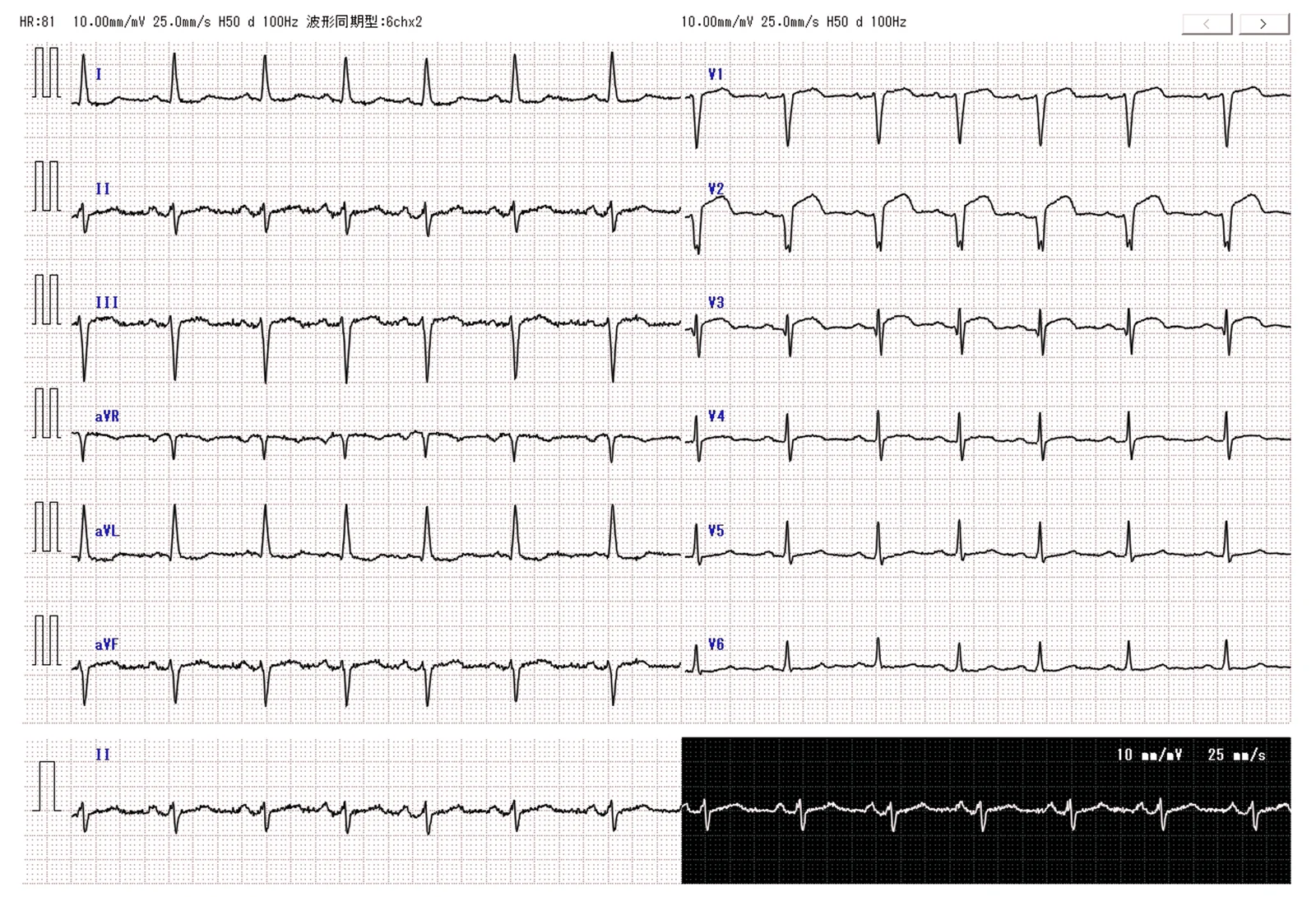
Electrocardiogram obtained before cardiac arrest. Electrocardiography showed QS complexes in leads V1 and V2 and ST-segment elevation in leads V1-V3.

A total of 27 min after arrival at our institution, while the initial evaluation and preparations for emergent coronary angiography were ongoing, she suddenly developed severe distress and subsequently progressed to cardiac arrest. The initial cardiac rhythm was pulseless electrical activity. Cardiopulmonary resuscitation was immediately initiated, with continuous chest compressions and intravenous epinephrine administered approximately every 4 min (Figure 2). Point-of-care transthoracic echocardiography revealed newly developed pericardial effusion that had not been present on the initial examination, strongly suggesting left ventricular free wall rupture (LVFWR) with cardiac tamponade. Venoarterial extracorporeal membrane oxygenation (VA-ECMO) was initiated as extracorporeal cardiopulmonary resuscitation through percutaneous cannulation of the right femoral artery with a 15-Fr arterial cannula and the right femoral vein with a 19.5-Fr venous cannula. The low-flow time from cardiac arrest to the initiation of extracorporeal support was 16 min. Because fluoroscopy was unavailable in the emergency department, we inserted both cannulas without fluoroscopic guidance and determined insertion depths using estimated surface anatomical measurements. After establishment of the extracorporeal circuit, venous drainage remained markedly impaired, presumably because of cardiac tamponade. Consequently, the extracorporeal blood flow was limited to 1.5 L/min. The patient was 148 cm tall and weighed 58 kg, with a body surface area of 1.51 m^2^. The target ECMO flow was calculated using a cardiac index of 2.6 L/min/m^2^ (2.6 L/min/m^2^ × 1.51 m^2^), resulting in a target flow of approximately 3.9 L/min. Thus, the achieved extracorporeal flow was substantially lower than the calculated full-flow requirement. Despite the limited extracorporeal flow, the patient was transferred immediately to the operating room for definitive surgical treatment.

**Figure 2:**
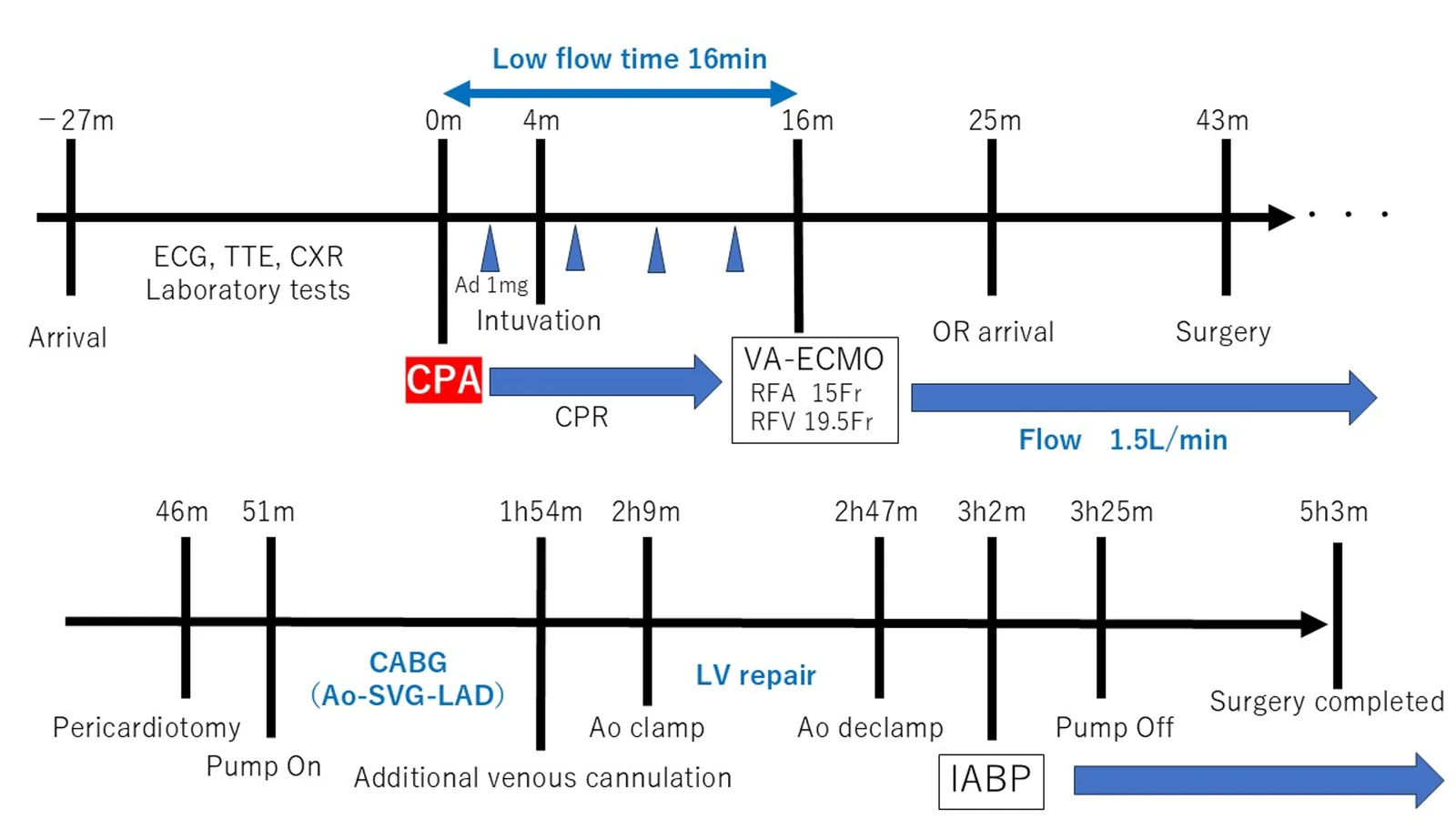
Timeline from cardiac arrest to completion of surgery. Cardiopulmonary resuscitation (CPR) was initiated immediately after cardiac arrest and discontinued after the establishment of venoarterial extracorporeal membrane oxygenation (VA-ECMO). The patient was promptly transferred to the operating room and transitioned from VA-ECMO to cardiopulmonary bypass (CPB) 51 min after entering the operating room. An additional 26-Fr venous cannula was inserted 1 h 54 min later to improve venous drainage during CPB. Coronary artery bypass grafting to the left anterior descending artery was performed on the beating heart under CPB. During this period, bleeding from the left ventricular rupture continued, but the shed blood was suctioned and returned to the CPB circuit. For definitive repair of the left ventricular rupture, the ascending aorta was cross-clamped at 2 h 9 min, and cardiac arrest was induced using Miotecter cardioplegic solution. The aortic cross-clamp was released 2 h 47 min after completion of the repair. An intra-aortic balloon pump (IABP) was inserted intraoperatively, and the patient was subsequently weaned from CPB with IABP support. The operation was then completed. The aortic cross-clamp time was 38 min, the total CPB time was 2 h 37 min, and the total operative time was 4 h 23 min. This image was created by the authors of this study using Microsoft PowerPoint software (Redmond, WA: Microsoft Corp.). Ao-SVG-LAD: aorta-saphenous vein graft-left anterior descending artery; TTE: transthoracic echocardiography; CXR: chest x-ray; CPA: cardiopulmonary arrest; CABG: coronary artery bypass grafting; LV: left ventricle; RFA: right femoral artery; RFV: right femoral vein

Surgery was initiated 1 h and 10 min after arrival at our institution and 43 min after the onset of cardiac arrest (Figure 2). A median sternotomy was performed. Upon opening the pericardium, a large amount of hematoma was evacuated. The anterior wall of the left ventricle was markedly aneurysmal, and pulsatile bleeding was observed from the inferior aspect of the aneurysmal region. The femoral VA-ECMO circuit was converted to cardiopulmonary bypass (CPB). Because adequate pump flow could not be achieved using the existing femoral venous cannula alone, an additional 26-Fr venous cannula was inserted directly into the right atrium, thereby allowing full-flow CPB to be established. Based on the electrocardiographic findings, an acute myocardial infarction involving the LAD territory was clinically suspected; however, the culprit coronary lesion could not be definitively identified because coronary angiography was not performed. Intraoperatively, the LAD was identified, and direct epicardial ultrasonography demonstrated blood flow within the vessel. The role of concomitant coronary artery bypass grafting during surgical repair of postinfarction LVFWR remains controversial, and a clear survival benefit has not been established. However, revascularization is generally considered reasonable when significant coronary disease or an infarct-related vessel can be addressed without delaying definitive repair. In the present case, because coronary angiography could not be performed before surgery, the exact culprit lesion remained unconfirmed; nevertheless, the electrocardiographic findings strongly suggested an LAD-territory infarction. Therefore, concomitant revascularization of the LAD was performed to provide reliable blood supply to the suspected infarct-related territory. Single-vessel coronary artery bypass grafting using an aorta-saphenous vein graft-LAD configuration was performed on the beating heart. The saphenous vein was harvested from the left lower leg.

Persistent pulsatile bleeding was subsequently identified at the inferoapical wall of the left ventricle within the aneurysmal region, where a macroscopically visible full-thickness myocardial defect communicating with the left ventricular cavity was directly identified, confirming a blow-out-type left ventricular free wall rupture (Figures 3A, 3B). Because of the ongoing bleeding and marked fragility of the infarcted myocardium, the repair was performed under cardiac arrest induced with Miotecter cardioplegic solution. After mobilization of the heart, inspection of the inferoapical rupture site revealed the full-thickness blow-out defect, surrounded by aneurysmally degenerated and extensively infarcted myocardium. Two 15-mm-wide polytetrafluoroethylene (PTFE) felt strips were placed on either side of the rupture so that the infarcted myocardium was securely sandwiched between them. The rupture was closed with four U-shaped horizontal mattress sutures using 3-0 polypropylene sutures with MH needles (Figure 3C). An additional PTFE felt strip was then placed over the repair site between the two lateral PTFE felt strips, and the repair was further reinforced with two layers of continuous over-and-over sutures (Figure 3D). Complete hemostasis was achieved after completion of the felt-reinforced repair. Because the patient did not require high-flow mechanical circulatory support after definitive repair and revascularization, an intra-aortic balloon pump (IABP) was selected to provide modest left ventricular afterload reduction and augment diastolic coronary perfusion during the early postrepair period. The patient was smoothly weaned from CPB with IABP support and only minimal inotropic support with dobutamine. Because no additional high-flow mechanical circulatory support was required after successful weaning from CPB, VA-ECMO was discontinued, and the femoral arterial and venous cannulas were removed intraoperatively. Transesophageal echocardiography after weaning from CPB showed no significant reduction in left ventricular cavity size and only mild mitral regurgitation, with no other clinically significant abnormalities.

**Figure 3:**
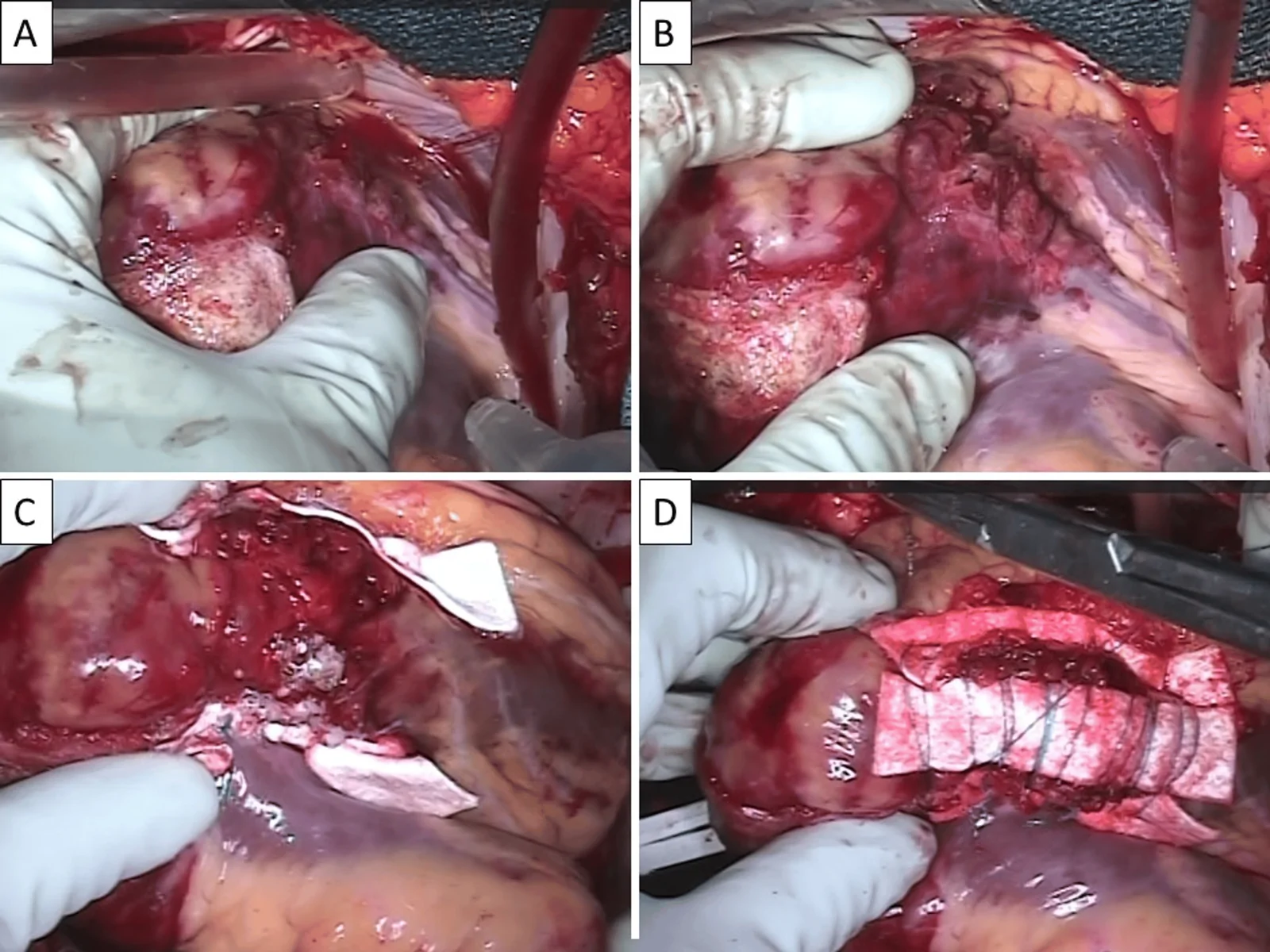
Intraoperative identification of the left ventricular rupture site and repair using felt strips. (A) A left ventricular free wall rupture with pulsatile bleeding was identified in the inferior wall of the left ventricle. (B) Dark-red discoloration of the myocardium was observed around the rupture site, suggesting extensive myocardial infarction. (C) The infarcted myocardium was sandwiched between two 15-mm-wide felt strips and closed with four interrupted U-shaped mattress sutures using 3-0 polypropylene sutures with MH needles. (D) An additional felt strip was placed over the repair site between the two felt strips, and the repair was reinforced with two layers of continuous over-and-over sutures.

Postoperatively, the patient was admitted to the intensive care unit (ICU) under deep sedation, mechanical ventilation, and IABP support. There was initial concern regarding hypoxic-ischemic neurological injury because extracorporeal blood flow had remained markedly limited before surgical decompression. However, on postoperative day one, she demonstrated purposeful responses and was able to follow verbal commands. She was successfully weaned from IABP support on postoperative day two and was extubated on postoperative day three. Transthoracic echocardiography performed on postoperative day four demonstrated reduced left ventricular wall motion in the LAD territory, together with thinning and aneurysmal dilatation of the apex. Her hemodynamic and respiratory conditions remained stable, and she was discharged from the ICU on postoperative day seven. Despite her overall clinical improvement, she developed marked lower-extremity muscle weakness, which was attributed to perioperative immobilization and physical deconditioning. After continued rehabilitation and stabilization of her general condition, she was transferred to a rehabilitation hospital for further therapy. After recovery from cardiac arrest, brain computed tomography showed no evidence of cerebral infarction or other acute intracranial abnormalities. Following discharge from the intensive care unit, the patient demonstrated no clinically apparent neurological deficits and recovered to a level at which specialist neurological consultation was not considered necessary. Electroencephalography was not performed. During hospitalization, her neurological functional status was assessed by the rehabilitation staff as Cerebral Performance Category (CPC) 1. At the one-year postoperative follow-up, she remained clinically well, was able to walk independently, and continued regular outpatient follow-up, with no apparent neurological deterioration.

## Discussion

LVFWR is one of the most catastrophic mechanical complications of acute myocardial infarction. For surgical purposes, LVFWR is often categorized as either the blow-out type or the oozing type [1]. Blow-out-type rupture is characterized by a macroscopic, full-thickness myocardial defect with rapid intrapericardial hemorrhage, frequently resulting in cardiac tamponade, profound hemodynamic collapse, and cardiac arrest. In contrast, oozing-type rupture is characterized by bleeding from the infarcted myocardium without a macroscopically evident tear and may progress more gradually, occasionally permitting temporary hemodynamic stabilization before definitive treatment. Previous surgical studies have demonstrated substantially poorer outcomes in patients with blow-out-type LVFWR than in those with oozing-type rupture. Blow-out-type LVFWR therefore usually requires immediate surgical intervention. The present case is notable because the patient achieved neurologically intact survival after cardiac arrest caused by blow-out-type LVFWR in an emergency department where fluoroscopy was unavailable, despite markedly restricted extracorporeal blood flow before emergency surgical repair (Table 1) [2-4].

**Table 1:** Reported cases of blow-out-type left ventricular free wall rupture. Among the reported cases with favorable neurological outcomes, cardiac arrest occurred exclusively in the cardiac catheterization laboratory. This case may add to the limited literature describing neurologically intact survival after ECPR for blow-out-type left ventricular free wall rupture when extracorporeal support is initiated outside the catheterization laboratory without immediate fluoroscopic guidance. PEA: pulseless electrical activity; ECPR: extracorporeal cardiopulmonary resuscitation

Studies	Age/sex	Blow-out	Cardiac arrest	Location	Pericardiocentesis	Neurological outcome
Arai et al. (2020) [2]	79/M	Yes	PEA → asystole	Catheterization laboratory	Yes	No neurological deficits
Kato et al. (2023) [4]	65/M	Yes	PEA	Catheterization laboratory	Yes	Severe hypoxic brain injury
Tautz et al. (2026) [3]	65/M	Yes	PEA	Catheterization laboratory	Yes	No neurological deficits
Present case	74/F	Yes	PEA	ED	No	No neurological deficits

VA-ECMO may provide temporary circulatory support as a bridge to surgery in patients with refractory shock or cardiac arrest caused by LVFWR. However, its effectiveness may be substantially limited in the presence of cardiac tamponade. Elevated intrapericardial pressure impairs right-sided cardiac filling and systemic venous return, thereby reducing the volume of blood available for drainage through the venous cannula [5]. Consequently, increasing the pump speed may not produce a corresponding increase in extracorporeal blood flow and may instead generate excessive negative pressure within the drainage circuit. In the present case, the tip of the femoral venous drainage cannula was identified intraoperatively within the inferior vena cava. This position may have resulted in inadequate drainage from the superior vena cava. In the setting of cardiac tamponade, insufficient superior vena cava drainage could theoretically increase cerebral venous pressure and reduce the effective cerebral perfusion pressure, thereby further compromising cerebral blood flow despite ongoing extracorporeal support. Although cerebral venous pressure was not directly measured and this mechanism cannot be confirmed in our patient, the cannula position represents an additional factor that may have adversely affected cerebral perfusion during the restricted-flow period. Under such circumstances, VA-ECMO can provide only partial circulatory support and cannot correct the underlying mechanical cause of hemodynamic collapse. Restoration of adequate circulation therefore requires prompt relief of cardiac tamponade and surgical control of the ventricular rupture.

In the present case, point-of-care echocardiography performed immediately after cardiac arrest demonstrated new pericardial effusion that had not been present during the initial examination, raising immediate suspicion of LVFWR with cardiac tamponade. Extracorporeal cardiopulmonary resuscitation (ECPR) was initiated promptly; however, extracorporeal blood flow remained markedly restricted because of impaired venous drainage. If immediate sternotomy had not been feasible, cautious pericardial drainage might have been considered as a temporary bridge to improve venous return and extracorporeal circulation. Kato et al. reported a patient with blow-out-type LVFWR in whom a pericardial drainage catheter was connected to the venous drainage limb of the ECMO circuit to maintain extracorporeal circulation before surgery [4]. However, decompression of the pericardial space may reduce the temporary tamponade effect and exacerbate hemorrhage from the ventricular defect. Pericardial drainage should therefore be performed cautiously and only as a bridge to definitive surgical hemostasis. In the present case, pericardial drainage was not performed because immediate transfer to the operating room and surgical decompression through median sternotomy were feasible.

A particularly notable feature of this case was the favorable neurological outcome despite markedly restricted extracorporeal blood flow. Impaired venous drainage limited maximum VA-ECMO flow to approximately 1.5 L/min, which was substantially below the calculated target flow based on the patient’s body size. After initiation of VA-ECMO, chest compressions were discontinued, and the extracorporeal flow remained approximately 1.5 L/min for approximately 30 min until pericardial decompression and full-flow cardiopulmonary bypass were established. This restricted flow was unlikely to ensure normal systemic or cerebral perfusion. Nevertheless, it may have contributed to systemic oxygen delivery during the interval before definitive surgical circulation was established. The physiological adequacy of this support cannot, however, be determined from extracorporeal pump flow alone.

The favorable neurological outcome was probably multifactorial. The cardiac arrest was witnessed in the emergency department, cardiopulmonary resuscitation was initiated immediately, and the CPR-to-ECMO interval was limited to 16 min. Furthermore, the immediate availability of an ECMO-capable multidisciplinary team, an operating room, and cardiovascular surgical resources enabled rapid transition from ECPR to definitive surgical repair. Together, these factors may have minimized the duration and severity of systemic and cerebral hypoperfusion. The patient recovered with a Cerebral Performance Category score of 1, which was maintained at the one-year follow-up. However, because cerebral blood flow and cerebral oxygenation were not directly measured, the mechanisms underlying the favorable neurological recovery remain speculative.

Definitive surgical repair was essential despite the initiation of VA-ECMO. In the present case, the macroscopic ventricular defect, persistent pulsatile hemorrhage, and extensive fragility of the surrounding infarcted myocardium necessitated repair under cardiac arrest. We selected a felt-strip sandwich technique to distribute suture tension over a wider area of the fragile myocardium and achieve secure hemostasis. Because coronary angiography could not be performed before surgery and the preoperative findings strongly suggested extensive infarction in the left anterior descending (LAD) artery territory, concomitant bypass grafting to the LAD was performed to ensure revascularization of the infarct-related territory. An intra-aortic balloon pump was subsequently used to facilitate left ventricular unloading and provide additional hemodynamic support.

This case suggests that failure to achieve the calculated target extracorporeal flow should not automatically be equated with futility of ECPR. Restricted VA-ECMO flow may provide temporary circulatory support during rapid transfer to definitive surgical treatment. In the present case, electrical cardiac activity persisted on electrocardiography during VA-ECMO support; however, the pulse pressure was minimal, suggesting that effective native cardiac output was markedly limited. Because of the emergent circumstances and the need for immediate transfer to the operating room, detailed hemodynamic and metabolic parameters, including serial arterial blood gases and lactate levels, were not available during this brief period of restricted ECMO flow.

Therefore, the observed neurological recovery cannot be used to establish that an ECMO flow of approximately 1.5 L/min provided adequate systemic or cerebral perfusion. Rather, this case suggests only that even markedly restricted VA-ECMO flow may serve as temporary circulatory support during rapid transfer to definitive surgical treatment in selected circumstances. When LVFWR with cardiac tamponade is suspected, prolonged attempts to optimize ECMO flow should not delay surgical decompression and hemostasis. VA-ECMO should be regarded not as definitive treatment, but as a temporary bridge to immediate surgical repair.

Several limitations of this report should be acknowledged. This report describes a single patient, and neither the minimum extracorporeal flow required to maintain organ perfusion nor the maximum tolerable duration of restricted flow can be inferred from this outcome. Cerebral blood flow and oxygenation were not directly assessed, and the relative contributions of VA-ECMO and native cardiac output to systemic perfusion could not be determined. Furthermore, because the cannulas were inserted without fluoroscopic guidance, suboptimal venous cannula positioning could not be completely excluded as a contributor to impaired drainage. The favorable outcome in this patient should therefore not be used to define a generally acceptable flow threshold or management strategy.

## Conclusions

Blow-out-type LVFWR should be suspected when a patient with acute myocardial infarction develops sudden cardiac arrest and a newly appearing pericardial effusion. Cardiac tamponade may severely restrict venous drainage and extracorporeal blood flow during VA-ECMO. Even when the calculated target flow cannot be achieved, restricted extracorporeal support may still serve as a temporary bridge to definitive surgery; however, attempts to optimize ECMO flow should not delay surgical decompression and hemostasis. This case underscores the importance of immediate cardiopulmonary resuscitation, prompt echocardiographic assessment, rapid initiation of extracorporeal cardiopulmonary resuscitation, and early transition to definitive surgical repair.
